# Characterization, Purification of Poncirin from Edible *Citrus* Ougan (*Citrus reticulate* cv. Suavissima) and Its Growth Inhibitory Effect on Human Gastric Cancer Cells SGC-7901

**DOI:** 10.3390/ijms14058684

**Published:** 2013-04-24

**Authors:** Xiaoyan Zhu, Fenglei Luo, Yixiong Zheng, Jiukai Zhang, Jianzhen Huang, Chongde Sun, Xian Li, Kunsong Chen

**Affiliations:** 1 Laboratory of Fruit Quality Biology, Zhejiang University, Zijingang Campus, Hangzhou 310058, China; E-Mails: 20916075@zju.edu.cn (X.Z.); fenglei2012@zju.edu.cn (F.L.); zhjk2010@zju.edu.cn (J.Z.); xianli@zju.edu.cn (X.L.); akun@zju.edu.cn (K.C.); 2 The State Agriculture Ministry Laboratory of Horticultural Plant Growth, Development and Quality Improvement, Zhejiang University, Zijingang Campus, Hangzhou 310058, China; 3 Department of Surgery, Second Affiliated Hospital, School of Medicine, Zhejiang University, Hangzhou 310009, China; E-Mail: zyx_xxn@126.com; 4 Forestry Bureau of Ouhai, Wenzhou 325000, China; E-Mail: ohgxhjz@sina.com

**Keywords:** macroporous resin, high speed counter-current chromatograph (HSCCC), *Citrus reticulate*, poncirin, purification, gastric cancer

## Abstract

Poncirin is a bitter flavanone glycoside with various biological activities. Poncirin was isolated from four different tissues (flavedo, albedo, segment membrane, and juice sac) of Ougan fruit (*Citrus reticulate* cv. Suavissima). The highest content of poncirin was found in the albedo of Ougan fruit (1.37 mg/g DW). High speed counter-current chromatography (HSCCC) combined with D101 resin chromatography was utilized for the separation and purification of poncirin from the albedo of Ougan fruit. After this two-step purification, poncirin purity increased from 0.14% to 96.56%. The chemical structure of the purified poncirin was identified by both HPLC-PDA and LC-MS. Poncirin showed a significant *in vitro* inhibitory effect on the growth of the human gastric cancer cells, SGC-7901, in a dose-dependent manner. Thus, poncirin from Ougan fruit, may be beneficial for gastric cancer prevention. The purification method demonstrated here will be useful for further studies on the pharmacological mechanism of poncirin activity, as well as for guiding the consumption of Ougan fruit.

## 1. Introduction

Epidemiological studies indicate that fruit and vegetable consumption may prevent many chronic diseases such as cancer, diabetes and cardiovascular disease [1]. Fruits and vegetables contain large amounts of phenolic compounds and other active components, some of which show strong antioxidant activity and free radical scavenging capacity [2]. For example, onion fruit contains abundant phenolic compounds such as flavonoids and anthocyanins [3] and red berry *Vitis vinifera* is rich in flavonoid compounds including anthocyanins, flavonols and flavan-3-ols [4]. In citrus fruits, a high content of flavonoids were found in flavedo, albedo, segment membrane and juice sac; the flavonoid content and components varied with fruit varieties and tissues [5–7]; and these compounds showed high bioactivities such as antioxidant, anticancer, and hypoglycemic activity.

**Figure 1:**
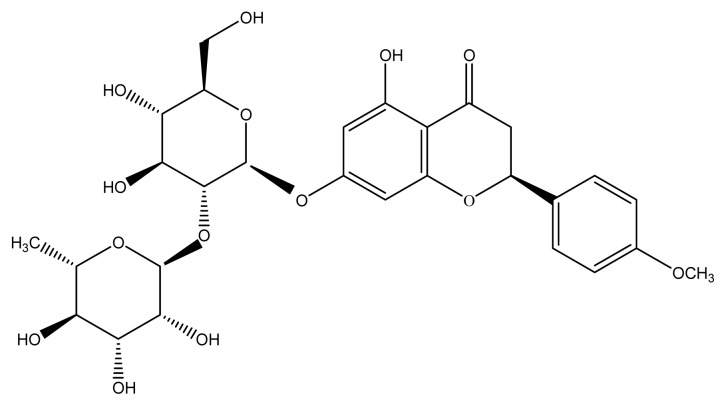
Molecular structure of poncirin.

Poncirin, the 7-*O*-neohesperidoside of isosakuranetin (Figure 1), is a flavanone glycoside with bitter taste. Pharmacological studies and clinical practices demonstrated that poncirin and its metabolites have multiple activities, such as anti-inflammatory properties [8], protective effects on potential gastric disease [9], defense against bacterial or viral infections and stresses [10], and promotion of osteoblast differentiation in mesenchymal stem cells [11].

Ougan (*Citrus reticulate* cv. Suavissima), also well known as Kugan, is the main mandarin cultivar local to Zhejiang province. Traditionally, Ougan fruit was listed as the royal tribute that lasted for centuries from the Song to the Qing Dynasty, and its high therapeutic effects had been recorded in the Compendium of Materia Medica and other herbal medicine references. Meanwhile, epidemiological and animal studies have also shown the close correlation between biological activities and the functional compounds of *Citrus* fruits [12]. Ougan fruit has been shown to have a wide variety of activities, such as anticarcinogenic activity, detoxification, and fever reducing [13].

Separation method by macroporous resins has been widely used in the separation and enrichment of bioactive compounds from many natural products for its simple procedure, high efficiency, low-cost, minor pollution and easy regeneration [14–17]. High-speed counter-current chromatography (HSCCC) is a support free liquid-liquid partition chromatography technique, which eliminates irreversible adsorption of samples onto a solid support matrix. HSCCC can yield a highly efficient separation of samples in several hours, so this method has been widely applied to the separation and purification of different kinds of natural products [18–20].

Gastric cancer, also called stomach cancer, is one of the diseases that have the highest mortality and morbidity worldwide, especially in Asia; it is the third most frequent cause of cancer death in China today. Thus, it would be significant to find potential anticancer dietary factors or antineoplastic drugs with potent therapeutic effects on gastric cancer. So far, poncirin has been reported to be possibly useful for treatment or protection of gastritis [9]. Crude extracts containing poncirin had inhibitory activity on human gastric cancer AGS cells [21]. However, to the best of our knowledge, the studies on the distribution of poncirin had only been seen in *Poncirus trifoliate* and *Citrus aurantium* [21,22]. Beyond that, few studies have focused on the distribution, bioactivity and purification analysis of poncirin in other fruit, especially in Ougan.

The objective of this study was to investigate the distribution of poncirin in different tissues of Ougan fruit, and to purify poncirin by a combination of macroporous resin column chromatography with HSCCC. In addition, the inhibitory effect of purified poncirin on the growth of gastric cancer SGC-7901 cells was also investigated for its potential function in gastric cancer prevention.

## 2. Results and Discussion

### 2.1. Content of the Poncirin in Four Tissues of Ougan

While few studies have examined the content of poncirin in *Citrus* fruits, a recent study demonstrated that the poncirin content in *Citrus aurantium* was 108.6 mg/kg [21]. In the present study, Ougan fruit was dissected into four different tissues, namely flavedo, albedo, segment membrane, and juice sac, and the poncirin content in the four parts was analyzed. HPLC results suggested that there was a significant difference in poncirin content among the four different tissues of Ougan fruit (Table 1). The highest content of poncirin was detected in the albedo of Ougan fruit, followed by segment membrane, flavedo, and juice sac. Based on these results, the poncirin content in the albedo of nine other edible cultivars was also characterized (data not shown). Among the cultivars tested, Ougan albedo contained the highest poncirin, therefore, it was chosen as the original material for poncirin purification.

### 2.2. Purification of Poncirin from Albedo of Ougan

In order to conduct an intensive study of poncirin, we took advantage of the high quality of poncirin found in Ougan fruit. We developed a simple and efficient method for the preparative isolation of poncirin from Ougan albedo, using a method that combined D101 macroporous resin column chromatography with HSCCC.

**Figure 2:**
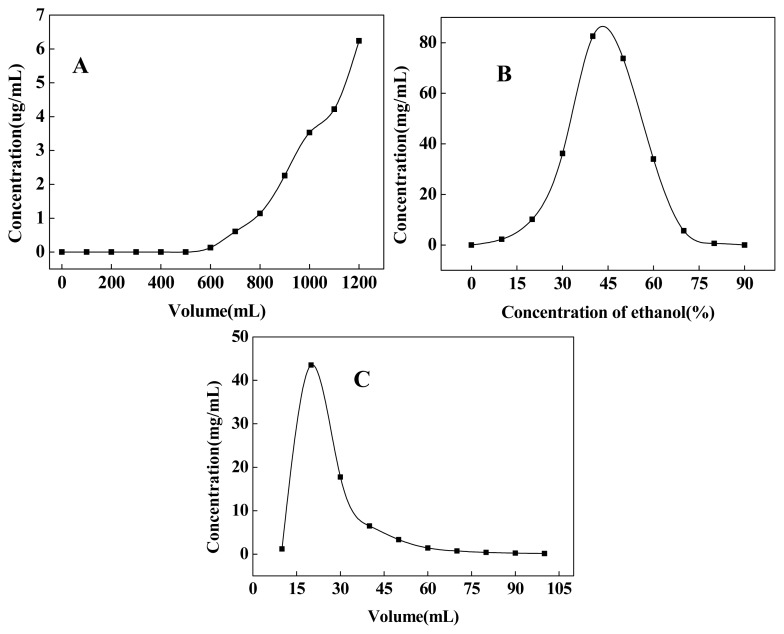
Dynamic leakage curves (**A**), dynamic desorption curves of poncirin by gradient elution (**B**), and isocratic elution (**C**) on column packed with D101 resin. Initial poncirin concentration in feed solution: 0.05 mg/mL; isocratic elution solution: 60% (*v*/*v*) ethanol; both adsorption and desorption flow rate: 1.0 mL/min.

The dynamic leakage curves on D101 resin were obtained for poncirin based on the volume of effluent and the original concentration of sample solution. As shown in Figure 2A, poncirin in solution was tightly absorbed by the macroporous resin before 500 mL. Then the concentration of poncirin in leak solution increased rapidly. In general, adsorption presumably reached saturation, when the concentration in effluent was 10% of the original concentration [23]. Therefore, 800 mL sample solution at the concentration of 0.05 mg/mL was selected as the feed volume on D101 resin for dynamic adsorption experiments in the present study (Figure 2A).

Dynamic desorption was carried out with gradient and isocratic elution modes at the flow rate of 1.0 mL/min, respectively. After loading 800 mL sample solution to the D101 resin column, gradient elution of poncirin was carried out by 3 BV (base volume) of each of different ethanol concentration (0%, 10%, 20%, 30%, 40%, 50%, 60%, 70%, 80% and 90%). The desorption ability of different solution changed together with the increased ethanol concentration and reached a maximum when the ethanol concentration was about 40% (Figure 2B). The majority of compounds absorbed by D101 resin were eluted by 10%–80% ethanol solutions (Figure 2B, Table 2). However, with the increase of ethanol concentration, the purity of poncirin decreased as more impurities with less polarity were desorbed. Considering both the purity and recovery of poncirin, 10% ethanol (16 BV) was selected to wash impurities and 60% ethanol (10 BV) was selected to elute target compound in isocratic elution experiments (Figure 2C). Under the optimized condition, all the effluent of 60% ethanol was collected and concentrated to dryness, which was used for further HSCCC purification.

A successful separation by HSCCC depends largely on the selection of a suitable two-phase solvent system, which provides an ideal partition coefficient (*K*, 0.5–2) for the targeted compound(s) [24,25]. As Ougan contains rich varieties of flavonoids, their separation is not an easy task. In this experiment, several kinds of solvent systems were tested, and the *K*-values for poncirin in these solvent systems were measured and summarized in Table 3. Among them, the two-phase solvent system, including ethyl acetate–*n*-butanol–water (4:0.5:5, *v*/*v*/*v*), ethyl acetate–*n*-butanol–water (5:1:5, *v*/*v*/*v*) had large *K*-values that tend to produce excessive sample band broadening when used for HSCCC separation. Likewise, chloroform–methanol–*n*-butanol–water (4:3:0.5:3, *v*/*v*/*v*/*v*) and n-hexane–*n*-butanol–water (1:1:2, *v*/*v*/*v*) that had small *K*-values resulted in poor peak resolution. Thus, these solvent systems were not suitable for the separation of poncirin from the resin-refined crude extract. At last, solvent systems of chloroform–methanol–*n*-butanol–water (4:3:0.5:2, *v*/*v*/*v*/*v*) resulted in ideal *K*-values and was chosen as the optimized HSCCC solvent system for poncirin purification.

**Figure 3:**
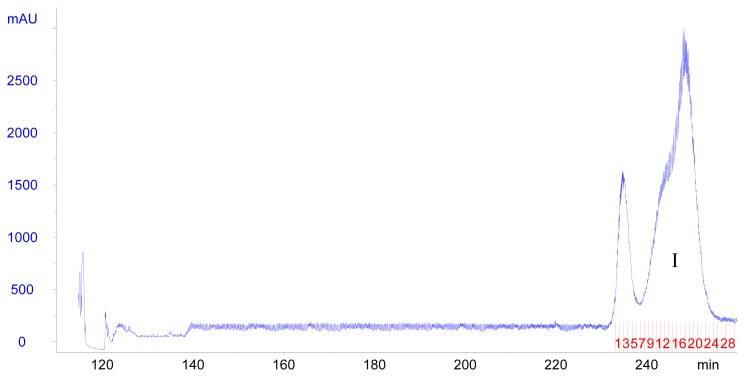
HSCCC separation chromatogram of poncirin. Solvent system: chloroform–methanol–*n*-butanol–water (4:3:0.5:2, *v*/*v*/*v*/*v*); flow rate (mobile phase): 2.0 mL/min; revolution speed: 850 rpm; sample size: 60 mg; detection wavelength: 280 nm. Fraction I was collected from tube # 8-27.

The influence of the flow rate of the mobile phase and the revolution speed of the separation column was also investigated. In our preliminary experiment, different flow rate (1.0, 2.0, 3.0 mL/min) of the mobile phase and different revolution speed (650, 750, 850, 950 rpm) of the selected system were examined. The result indicated that high flow rate of 3.0 mL/min was unfavorable to separate the target compound well. Low flow rate of 1.0 mL/min could produce a good separation, while it prolonged the separation time and extended the chromatogram peak. Though the high revolution speed of 950 rpm can increase the retention of the stationary phase, it also broadened the chromatogram peak and could not produce a good separation. Likewise, the use of a lower speed (650, 750 rpm) would lead to a lower peak resolution as it decreased the retention of the stationary phase. Considering these aspects, a flow rate of 2.0 mL/min and a revolution speed of 850 rpm were selected for poncirin purification in this study. Under the optimized conditions, the separation resulted in the collection of fraction I (tube # 8-27) to obtain pure poncirin (Figure 3).

**Figure 4:**
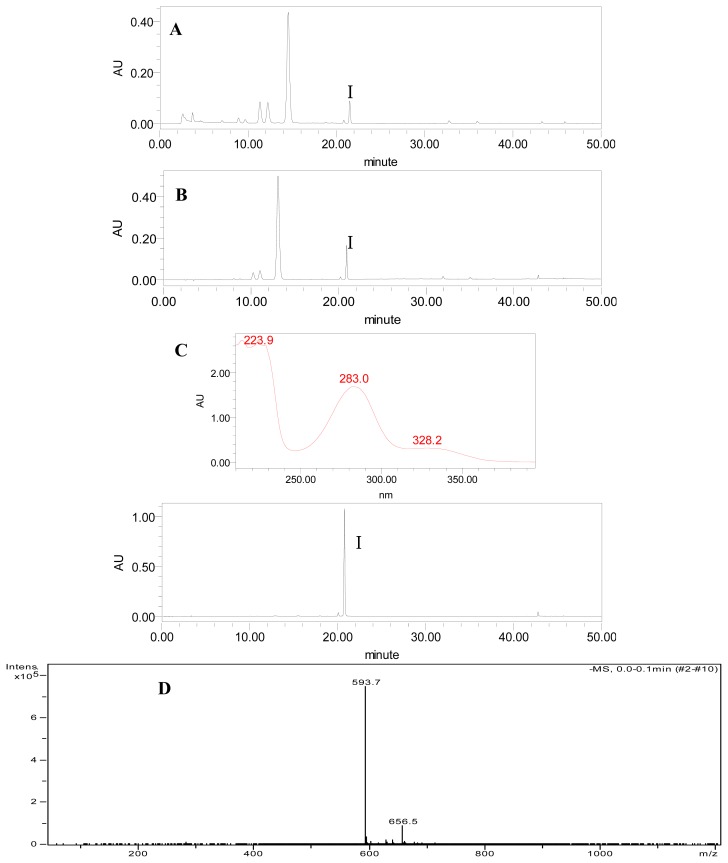

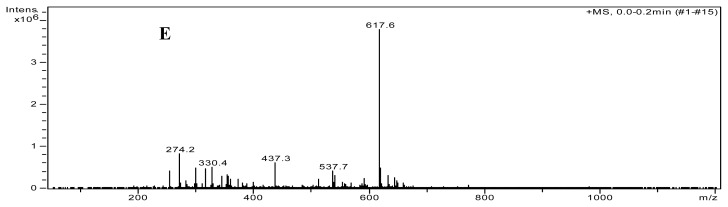
HPLC chromatogram and UV absorption spectrum of poncirin from crude extract before (**A**) and after (**B**) treatment with D101 resin and the purified product (**C**) from fraction I obtained by HSCCC. Peak I represents poncirin. (**D**) and (**E**), LC-MS chromatogram of HSCCC purified poncirin product.

The effect of the two-step purification was shown in Figure 4, and the purities and recoveries of poncirin in different procedures were summarized in Table 4. The purity of poncirin in the crude extract of Ougan albedo was as low as 0.14%. After one-step of D101 resin purification, the purity increased to 5.30%, which was 37.86-fold that of the crude extract. The poncirin recovery was 71.55% (Table 3). In addition, HSCCC purification produced 2.1 mg poncirin with 96.56% purity (Figure 4C) and the recovery rate was 63.77% from a 60 mg sample (Table 4). The structure of the isolated poncirin was further identified according to its UV and MS data. UV data obtained from HPLC-DAD analysis showed that the purified poncirin had the same retention time with that of the authentic standard (20.7 min), as well as the same maximum absorption peaks. Further structure identification of the purified poncirin was confirmed by LC-MS. The [M-H]^−^ ion at *m/z* 593.6 that had high abundance was observed in the purified poncirin, suggesting the molecular weight of poncirin might be 594.6 (Figure 4D). Additionally, typical fragment ions at *m/z* 330.4, 437, and 617.6 were detected in LC-MS^2^ chromatogram (Figure 4E). Compared with the mass spectrogram of the authentic standard, we can confirm ulteriorly that the purified compound was poncirin.

Although conventional methods including silica gel column or centrifugal partition chromatography had previously been developed to separate poncirin from *Poncirus trifoliate* [22], our methods that combined macroporous resin column chromatography with HSCCC, turned out to be more suitable due to its comparative high efficiency and excellent sample recovery.

### 2.3. Inhibitory Activity of Purified Poncirin on the Growth of Gastric Cancer Cells

**Figure 5:**
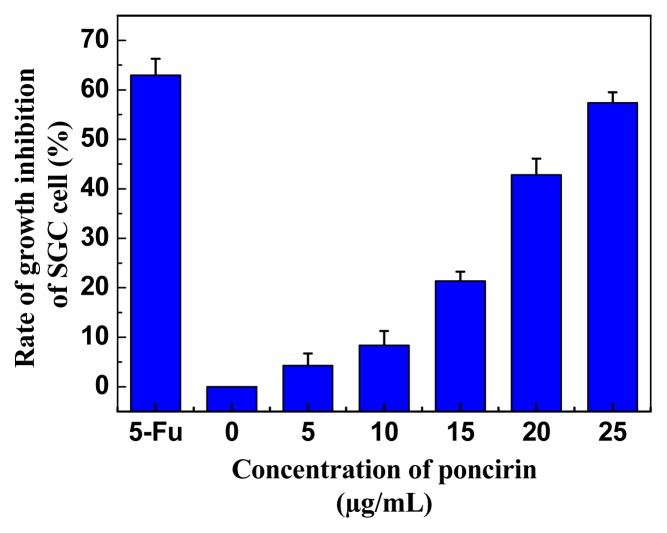
Inhibitory activity of purified poncirin on the growth of gastric cancer SGC-7901 cells. 5-Fu (positive control), 5-fluorouracil; Results are presented as mean ± S.D. (*n* = 9).

The gastric cell line SGC-7901 was used to test the effect of purified poncirin on cell proliferation. The result showed that purified poncirin could significantly inhibit the proliferation of human gastric cancer cells in a dose-dependent manner, and that growth inhibition reached 57.3% at the concentration of 25 μg/mL (Figure 5). The toxicity of poncirin had been evaluated and no toxicity was shown at the tested concentration scale from 5 to 25 μg/mL. The protective effect of *Poncirus trifoliate* on potential gastric disease was previously analyzed with rat gastritis and human gastric cancer cell models; results showed the protective effect was associated with constituents such as noehesperidin and poncirin [9]. Also, flavonoids isolated from *Citrus aurantium* had anti-proliferation effects on human gastric cancer AGS cells with an IC_50_ value of 99 μg/mL [21]. Yijin-tang (Nichin-to in Japanese and Er-chen-tang in Chinese), an oriental herbal formula, is composed of five different herbs, including *Citrus unshiu*, *Glycyrrhiza uralensis*, *Pinellia ternate*, *Poria cocos*, and *Zingiber officinale*. It can protect gastric mucosa from ethanol-induced acute gastric injury via increasing the antioxidant status with 1.5 × 10^−4^ mg/g poncirin [26]. Thus, poncirin had the potential to be used as preventive agents against human gastric cancer, and further studies are needed to explain the mechanism.

## 3. Experimental Section

### 3.1. Chemical and Reagents

Poncirin standard was purchased from ChromaDex Inc. (Irvine, California, CA, USA). D101 macroporous resin was bought from Bohong Resin Technology Co., Ltd. (Tianjin, China). Acetonitrile and methanol of chromatographic grade, dimethyl sulfoxide (DMSO) and methyl thiazolyl tetrazolium (MTT) were purchased from Sigma-Aldrich (St. Louis, MO, USA). Culture medium RPMI 1640 and fetal calf serum were purchased from Gibco (Invitrogen, China). All solvents used for extraction and HSCCC were of analytical grade and purchased from Sinopharm Chemical Reagent Co., Ltd. (Shanghai, China). Double-distilled water (ddH_2_O) was used for all solutions and dilutions. All solutions for HPLC analysis were filtered through 0.45 μm membrane before use.

### 3.2. Apparatus

The preparative HSCCC instrument employed in the present study was a TBE-300 high-speed counter-current chromatograph (Tauto Biotech, Shanghai, China) with a PTFE three multilayer coil separation columns (id. of the tubing, 1.6 mm; total column volume, 260 mL) and a 20 mL sample loop. The revolution radius or the distance between the holder axis and central axis of the centrifuge (R) was 5 cm, and the β-values of the multilayer coil varied from 0.5 (internal terminal) to 0.8 (external terminal). The revolving speed of HSCCC was adjustable, ranging from 0 to 1000 rpm. The experimental temperature was controlled by an HX 1050 constant temperature-circulating implement (Boyikang Lab Instrument Company, Beijing, China). An ÄKTA prime system (GE Healthcare, Uppsala, Sweden) was used to pump the two-phase solvent system and perform the UV absorbance measurements. The data were collected by a Unicorn 5.11 chromatography workstation.

The HPLC equipment used was Waters Alliance 2695 system (Waters Corp., Milford, CT, USA) and consisted of a 2996 photodiode array detector and an Empower chromatography workstation.

The LC-MS experiment was performed using a Bruker Esquire 3000 plus mass spectrometer (Bruker-Franzen Analytik GmbH, Bremen, Germany) equipped with an ESI source and ion trap analyzer in the negative-ion mode.

### 3.3. Materials

Ougan fruits were collected in November 2010 from Wenzhou, Zhejiang province, China. The fruits were separated into four parts, *i.e.*, flavedo, albedo, segment membrane and juice sac, and cut into small pieces respectively. The parts were dried at 50 °C in a drying oven, powdered and sieved with a 40 meshes sieve and stored at −40 °C until analysis.

### 3.4. Preparation of Sample Solution

Each part of Ougan fruit (0.5 g FW) was extracted twice with 10 mL 80% ethanol for 30 min respectively and both extracts were combined and evaporated to dryness at 50 °C. The residue was dissolved in 2 mL methanol and centrifuged at 10,000 rpm for 10 min. The supernatant was used for HPLC analysis.

Dry albedo powder (20 g) was extracted twice by sonication with 400 mL 80% ethanol. The extract was then evaporated to form a syrup by a rotary evaporator at 50 °C under reduced pressure and then dissolved in ddH_2_O. The water-soluble extract containing 0.05 mg/mL poncirin was then used for the separations in subsequent experiments.

### 3.5. Identification of Poncirin

The HPLC analysis was accomplished with a Waters C_18_ column (250 mm × 4.6 mm, I.D., 5 μm) at 25 °C. ddH_2_O (A)-acetonitrile (B) was used as the mobile phase in gradient elution mode, and the elution system was as follows: 0–10 min, 78% of A; 10–35 min, 78%–39% of A; 35–40 min, 39%–0 of A; 40–42 min, 0 of A; 42–45 min, 0–78% of A; 45–50 min, 78% of A. The flow rate was 1.0 mL/min and the detection wavelength was 280 nm.

For the LC-MS analysis, nitrogen was used as the nebulizing gas at a pressure of 10 psi and the drying gas at a flow rate of 5 L/min. The ion-source temperature was set at 250 °C, and the capillary voltage was set at 4000 V. Data reported here were acquired using software Esquire 5.0 (Bruker, Massachusetts, MA, USA).

### 3.6. Dynamic Adsorption and Desorption Tests

In the present study, D101 macroporous resin was selected to elute the target compound according to [27]. Before use, D101 macroporous resin was soaked with 95% aqueous ethanol for 24 h and then washed with 5% HCl, 5% NaOH, and ddH_2_O successively.

Dynamic adsorption and desorption experiments were carried out with a glass column (Ø16 × 200 mm, Shanghai Qite Analytical Instrumental Co., Ltd., Shanghai, China) wet-packed with D101 macroporous resin. The bed volume (BV) of resin was 10 mL and sample solution flowed through the glass column at the flow rate of 1.0 mL/min. The concentration of poncirin in the sample solution used in our experiment was 0.05 mg/mL, and the concentrations of poncirin in different effluents were monitored by HPLC analysis in order to determine the feed volume. For desorption experiment, the column was first washed with 3 BV (30 mL) water after reaching adsorptive saturation, and then eluted by different concentrations of ethanol-water solution. The concentration of poncirin in each of the effluents was monitored by HPLC analysis, and then concentrated to dryness under vacuum.

### 3.7. Selection of the Two-Phase Solvent Systems for HSCCC

The two-phase solvent system was selected according to the partition coefficient (*K*) of the target compounds. The *K*-value was determined by HPLC as follows: suitable amount of resin-refined sample was dissolved in different pre-equilibrated solvent systems (upper phase/lower phase, 1:1, *v*/*v*) and mixed thoroughly. After the equilibration was reached, both the upper phase and the lower phase were analyzed for the target compound and their HPLC peak areas were recorded as *A*1 and *A*2, respectively. The *K*-value was calculated according to the following equation: *K* = *A*1/*A*2.

### 3.8. Preparation of Two-Phase-Solvent System and Sample Solution

In the present study, the two-phase solvent system of chloroform–methanol–*n*-butanol–water (4:3:0.5:2, *v*/*v*/*v*/*v*) was selected for HSCCC separation. Each component of the solvent system was added into a separating funnel and thoroughly equilibrated overnight. The upper and lower phases were separated, and degassed by ultrasonic for 30 min before use. Resin-refined sample (60 mg) was prepared by dissolving the sample in mix solution of 3 mL upper and 3 mL lower phases.

### 3.9. HSCCC Separation Procedure

In HSCCC separation, the multiplayer coiled column was first filled with the upper phase at a flow rate of 15 mL/min. The apparatus was then rotated at 850 rpm, and the lower phase was pumped into the column from head to tail at a flow rate of 2 mL/min. After the mobile phase front emerged and hydrodynamic equilibrium was established, 6 mL sample solution was injected. The effluent of the column was monitored by a UV detector at 280 nm and the separation temperature was set at 25 °C. Peak fractions were collected (1.8 mL/tube) according to the elution profile for HPLC, LC-MS analysis and cell viability assay.

### 3.10. Cell Culture and Cell Viability Assay

The human gastric cancer cell line SGC-7901 was cultured in RPMI 1640 medium supplemented with heat inactivated fetal bovine serum (FBS, 10%), penicillin (100 U/mL), and streptomycin (100 μg/mL). The cells were grown at 37 °C in an incubator containing 5% CO_2_. Exponentially growing cells were used for experimentation. The inhibitory effect of the purified poncirin on human gastric cancer SGC-7901 cells was determined by cell viability assay performed with MTT photometric analysis as described by Mosmann with some modifications [28]. Briefly, cells were seeded in 96-well plates at a density of 6000 cells/well in 200 μL of medium. With appropriate adhesion, the supernatant was removed and the cells were treated with 200 μL of medium containing 0 (control), 1, 2, 3, 4, and 5 μg purified poncirin, respectively. Cells treated with commercial anticarcinogen 5-fluorouracil (5-Fu) of 30 μg/mL were set as positive control. Each treatment was conducted in six wells. Forty-eight hours after treatment, 20 μL MTT solutions (5 mg/mL) were supplemented for each well. The plate was shaken evenly and then incubated for 4 h at 37 °C. The MTT solution was removed carefully after incubation, and 200 μL/well of DMSO was added. The plates were shaken for 10 min in a balance oscillator, and the absorbance (*A*) was measured at 490 nm by a plate reader. The inhibition rate (*IR*) of cell proliferation was calculated as follows: *IR* (%) = (*A*_control_ − *A*_treatment_)/*A*_control_ × 100%.

### 3.11. Statistic Analysis

Experiments were performed in triplicate and data were expressed as the mean ± standard deviation.

## 4. Conclusions

In the present study, poncirin in Ougan fruit was analyzed and purified. Ougan albedo was found to be a good resource of poncirin among edible citrus fruit. HSCCC combined with D101 macroporous resin is efficient in preparative isolation of poncirin from Ougan fruit, with poncirin purity increased from 0.14% to 96.56% after the two-step purification. Purified poncirin was identified by HPLC-PDA and LC-MS, and it showed a significant *in vitro* inhibitory effect on the growth of human gastric cancer cells SGC-7901 in a dose-dependent manner; thus poncirin is potentially beneficial for patients that suffer from stomach cancer. The established purification method is useful for guiding the consumption of Ougan fruit as well as for further studies on the pharmacological mechanism of poncirin.

## Figures and Tables

**Table 1:** Content of the poncirin in four tissues of Ougan fruit.

Tissue	Content of poncirin (mg/g FW)
flavedo	0.15 ± 0.01
albedo	1.37 ± 0.09
segment membrane	0.52 ± 0.01
juice sac	0.07 ± 0.01

Results are presented as mean ± S.D. (*n* = 3) on a fresh weight (FW) basis.

**Table 2:** Results of gradient elution of the poncirin on column packed with D101 resin.

Concentration of ethanol (%)	Mass of dried residue (mg)	Mass of the poncirin (mg)	Content of the (poncirin %)
10	19.3	/	/
20	140.5	0.78	0.56
30	217.4	3.91	1.80
40	193.5	9.48	4.90
50	82.9	8.42	10.16
60	38.3	3.65	9.52
70	13.7	0.24	1.76
80	2.3	/	/
90	0.7	/	/

**Table 3:** The partition coefficients (*K*) of the poncirin in different solvent systems of HSCCC. The *K* value was defined as the peak area of the compound in the upper phase divided by that in the lower phase.

Solvent system	Ratio(*v*/*v*/*v*/*v*)	*K* value
Chloroform–methanol–*n*-butanol–water	4:3:0.5:2	0.81
Chloroform–methanol–*n*-butanol–water	4:3:0.5:3	0.36
Ethyl acetate–*n*-butanol–water	4:0.5:5	3.14
Ethyl acetate–*n*-butanol–water	5:1:5	2.73
*n*-Hexane–*n*-butanol–water	1:1:2	0.55

**Table 4:** The purities and recoveries of poncirin in the two-step purification.

Purification step	Purity (%)	Recovery (%)	Yield (mg)
Crude extract	0.14	/	/
D101 Resin	5.30	71.55	302.4 a
HSCCC	96.56	63.77	2.1 b

a The amount of the refined sample was obtained from 16 g raw material in the resin purification;

b The amount of compound was obtained from 60 mg refined sample by one HSCCC run.
